# The Impact of the National Essential Medicines Policy on Rational Drug Use in Primary Care Institutions in Jiangsu Province of China

**Published:** 2018-01

**Authors:** Jianqian CHAO, Jiangyi GU, Hua ZHANG, Huanghui CHEN, Zhenchun WU

**Affiliations:** Key Laboratory of Environmental Medicine Engineering of Ministry of Education, School of Public Health, Southeast University, Nanjing, Jiangsu, China

**Keywords:** Primary care institutions, Rational drug use, National essential medicines policy, China

## Abstract

**Background:**

Essential medicine policy is a successful global health policy to promote rational drug use. The aim of this study was to evaluate the impact of the National Essential Medicines Policy (NEMP) on the rational drug use in primary care institutions in Jiangsu Province of China.

**Methods:**

In this exploratory study, a multistage, stratified, random sampling was used to select 3400 prescriptions from 17 primary care institutions who implemented the NEMP before (Jan 2010) and after the implementation of the NEMP (Jan 2014). The analyses were performed in SPSS 18.0 and SPSS Clementine client.

**Results:**

After the implementation of the NEMP, the percentage of prescribed EML (Essential Medicines List) drugs rose significantly, the average number of drugs per prescription and average cost per prescription were declined significantly, while the differences of the prescription proportion of antibiotics and injection were not statistically significant. BP (Back Propagation) neural network analysis showed that the average number of drugs per prescription, the number of using antibiotics and hormone, regional differences, size of institutions, sponsorship, financial income of institutions, doctor degree, outpatient and emergency visits person times were important factors affecting the prescription costs, among these the average number of drugs per prescription has the greatest effect.

**Conclusion:**

The NEMP can promote the rational use of drugs in some degree, but its role is limited. We should not focus only on the EML but also make comprehensive NEMP.

## Introduction

The medically appropriate use of drugs is not only the measure for prevention and cure diseases but also the important guarantee for the quality of medical services and the rational use of medical resources (1). However, inadequate use of medications was commonly observed. More than 50% of all drugs were prescribed, dispensed or sold inappropriately in the world, and 50% patients could not use drugs correctly. About 1/3 deaths in the world died of irrational use of drugs including hyper medication and faulty drugs (2). More than 60% of the patients’ prescriptions violated the standard treatment guidelines in the basic health system of developing and transition countries. The irrational use of drugs in China was relatively serious, polypharmacy, abuse of antibiotics and injection, were common, the frequency and proportion of prescribed antibiotics were higher compared with the developed countries (3, 4).

Essential medicine policy is a successful global health policy to promote rational drug use (5). Some measures have been implemented to improve the rational use of drugs worldwide, such as in Delhi, Australia (6, 7).

In Aug 2009, the China State Department issued “the implementation opinions about establishing a National Essential Medicines Policy”, “National list of essential medicines management method (provisional)” and “the National list of essential medicines (equipping and using in primary health institutions)”, these marked that the China launch to establish the NEMP in whole. The rational use of drugs is one of the aims of the NEMP in China (8). Many researchers worldwide have evaluated the effect of different intervention measures for the rational use of drugs (9–11). The national practice essential medicine policy had important role in promoting rational drug use. However, the most research of the national medicine policy was cross-sectional design without control, it could not provide powerful evidence for intervention effect.

Scholars in China have also studied the relationship between the NEMP and rational use of drugs. The impact of the NEMP was assessed on the use of medicines in government-owned primary care institutions. It showed the NEMP interventions reduced the average cost per prescription, the irrational use of antibiotics and unnecessary parenteral administration remained, they did not study the factors affecting the rational drug use (12). At present, the empirical research on essential medicine policy and rational use of drugs in primary care institutions are less, and the research conclusions are inconsistent (13, 14).

Our study aimed to analyze the rational use of drugs, costs and influencing factors in primary care institutions, and then evaluate the effects of the NEMP on the rational use of drugs from the perspective of the empirical study through investigating the primary care institutions in Jiangsu Province.

## Methods

### Data sources and research design

This exploratory study was conducted in Jiangsu province in 2014. Jiangsu, one of the most developed provinces in China, is located in southeastern China, have thirteen cities. Because it was generally divided into three prefectures of Jiangsu to their levels of regional economic development, so three cities of Jiangsu Province were chosen, economic developed city A, moderately developed city B and economic undeveloped city C based on per capita GDP level of the pilot regions in Jiangsu province by using the method of stratified random sampling. Seven community health service institutions from A city, 5 from B and C city were selected to the health institutions proportionality, the total 17.

A questionnaire designed by the investigators was used to collect each community health service agencies prescriptions before (Jan 2010) and after (Jan 2014) the implementation of the NEMP, Numbering outpatient prescriptions in Jan 2010 and Jan 2014 of each agency to the dates. The first prescription was selected randomly, every five prescription extract one to 200 prescriptions (every 100 prescriptions for before and after the implementation of the NEMP). Overall, 3400 prescriptions were selected from 17 community health service agencies. The exclusion criteria were prescription writing unclear, drug names unidentifiable; lack of cost price; prescription of Chinese herbal medicine. Overall, 197 prescriptions were eliminated, 3203 prescriptions were examined. We have developed a strict quality control program in the design of the investigation, the implementation of the investigation and statistical analysis. Experts in the field were invited to discuss the design of the questionnaire, including the need for elimination of inappropriate items. Before the investigation, a pilot study was conducted on a small sample size. The questionnaire was further improved to the results. This process ensured the validity and reliability of the questionnaire.

### Data analysis

According to WHO Core Drug Use Indicators for the rational use of drugs in developing country (15) and researches in China (16), the indicators of this study include 1) percentage of drugs prescribed from the EML; 2) average number of drugs per prescription; 3) the prescription percentage of antibiotics; 4) the prescription percentage of injections (not containing prevention injection or plans immune); 5) average cost per prescription.

The data were entered into Excel 2007. The analyses were performed in SPSS 18.0 (SPSS Inc., Chicago, IL, USA) and SPSS Clementine client 11.0 software, with 0.05 set as the required level of significance.

Multiple regression analysis is commonly used in analyzing influencing factors, but the relationship between the average cost per prescription and its affecting factors is complicated and is not suitable for the multiple regressions. BP neural network is applicable to any data and its fitting function of data is better than multiple regression analysis. Nevertheless, BP neural network model cannot select variables automatically, so that no significant variables could be included in the model if all the independent variables in screening table were included in model to fit, it could reduce the efficiency of the model. We use multiple linear regression analysis to screen variable and then use BP neural network to analyze the influencing factors (17,18). The possible independent variables affecting the average cost per prescription are divided into seven aspects:

1. Variables reflecting the local socio-economic level (including geographical distribution and administrative region);
2. Variables reflecting the size of the organization (business occupancy area);
3. Variables reflecting the organization system construction (the sponsorship, whether carrying out the two lines of income and payment, whether being in fixed unit of medical insurance;
4. Variables reflecting construction of organization talent team (the proportion of the bachelor degree and above, the proportion of intermediate professional titles and above, the proportion of the personnel accepting general practitioners standardized training, the proportion of the personnel accepting the general practitioners post training and the personnel passing national general medicine intermediate technical qualifications) ;
5. Variables reflecting agency financial revenues and expenditures (business income);
6. Variables reflecting service delivery (outpatient and emergency visit person times;
7. Variables reflecting prescription medication (whether the prescription using intravenous, hormone, antibiotic and two or more antibiotics union, the averages number of prescription drugs) (Table 1).

In BP neural network via network testing, the model has the best fitting result when 60% samples by randomized selecting for network training and 20% samples for validation, 20% samples for model test of the results.

**Table 1:** Comparison of average number of drugs per prescription before and after the implementation of the NEMP

***Region***	***Time***	***Numbers of prescriptions***	***Max/min value***	***Average number of drugs per prescription***	***Standard deviation***	***Z***	**P**
City a	Jan 2010	100	1/7	3.254	1.609	−5.576	0.000
	Jan 2014	100	1/8	2.256	1.334		
City b	Jan 2010	100	1/10	3.211	1.473	−3.143	0.002
	Jan 2014	100	1/11	2.890	1.677		
City c	Jan 2010	100	1/14	3.890	2.887	−3.734	0.000
	Jan 2014	100	1/12	3.234	2.345		

### Ethical approval

This study obtained the approval by the Medical Ethics Committee of Southeast University. The study objectives and design were explained.

## Results

### Rational drug use in primary care institutions Percentage of drugs prescribed from the EML

The percentage of prescribed EML drugs in A, B, C city was 49.14%, 59.34%, 56.14%, respectively, before the NEMP interventions, the percentage of prescribed EML drug were 89.81%, 100%, 100% after NEMP implementation, it significantly rose (*P*<0.05).

### Average number of drugs per prescription

In order to avoid the effects of different month disease kinds, different annual and same month (Jan 2010 and Jan 2014) outpatients’ records were selected to analyze the changes of an average number of drugs per prescription before and after the implementation of the NEMP. Because of an average number of drugs per prescription not meeting the normal distribution, Mann-Whitney Test was used. Differences of average numbers of drugs per prescription before and after implementation of the NEMP were statistically significant, the average numbers of drugs per prescription in these three cities all declined after the implementation of the NEMP (Table 1).

### The prescription percentage of antibiotics

The prescription percentage of antibiotics is an important indicator for rational drug use assessed. There were no differences before and after the implementation of the NEMP in three cities (Table 2).

**Table 2:** Comparison of the prescription percentage of antibiotics before and after the implementation of the NEMP

***Region***	***Time***	***Percentage (%)***	***χ^2^***	***P***
City A	Jan 2010	42.30	2.219	0.061
	Jan 2014	44.45		
City B	Jan 2010	40.19	0.278	0.614
	Jan 2014	42.30		
City C	Jan 2010	39.34	0.034	0.854
	Jan 2014	37.65		

### The prescription percentage of injections

The prescription percentage of injections is also an important indicator for rational drug use assessed. Percentage of patients prescribed injections in three cities did not improve (Table 3).

**Table 3:** Comparison of the prescription percentage of injections before and after the implementation of the NEMP

***Region***	***Time***	***Percentage (%)***	***χ^2^***	***P***
City A	Jan 2010	24.34	2.422	0.120
	Jan 2014	30.28		
City B	Jan 2010	29.51	0.001	0.880
	Jan 2014	29.60		
City C	Jan 2010	25.45	2.322	0.134
	Jan 2014	24.40		

### The average cost per prescription

The average cost per prescription in three cities after the implementation was significantly declined (Table 4).

**Table 4:** Comparison of the average cost per prescription before and after the implementation of **t**he NEMP

***Region***	***Time***	***Average cost per prescription***	***T***	***P***
City A	Jan 2010	105.54	8.223	0.000
	Jan 2014	60.10		
City B	Jan 2010	101.89	4.218	0.000
	Jan 2014	61.56		
City C	Jan 2010	82.97	9.123	0.000
	Jan 2014	44.72		

### Factors that affecting the rational drug use

Because the prescription cost is the key index of rational use of drugs, we analyzed the factors affecting the average cost per prescription. The average cost of prescription was as the dependent variable and factors that may affect the amount of cost as the independent variables.

Firstly, we screened the variables using multiple regression analysis. Eleven variables were included in BP neural network: geographical distribution (X1), size of organization (X3), sponsorship (X4), proportion of bachelor degree and above (X7), proportion of intermediate professional titles and above (X8), the business income (X12), outpatient and emergency visit person times (X13), whether the prescription using antibiotics (X15), whether the prescription using hormone (X16), whether the prescription using two or more antibiotics union (X17), the average amount of prescription drugs (X18).

The determined coefficient of BP neural network model was 0.952 analyzed by single-layer network analysis. Eleven variables are included in the model: variables reflect regional differences of average number of drugs per prescription (differences among economic developed city A, moderately developed city B, economic undeveloped city C); size of organization; sponsorship; primary care supplier such as proportion of bachelor degree and above; proportion of intermediate professional titles and above; the agency financial revenues and expenditures; delivery service and prescription drugs like using antibiotics, hormone and two or more antibiotics union. The sensitivity analysis results showed that average number of drugs per prescription has the greatest impact on prescription costs (Table 5).

**Table 5:** The influencing factors analyzed by BP neural network

***Variable***	***Sensitivity***
Average number of drugs per prescription	0.215
Proportion of intermediate professional titles and above	0.195
Business income	0.086
Geographical distribution	0.062
Proportion of bachelor degree and above	0.050
Outpatient and emergency visits person times	0.050
Sponsorship	0.044
Whether the prescription using antibiotics	0.016
Whether the prescription using hormone	0.009
Whether the prescription using two or more antibiotics union	0.007
Size of organization	0.001

## Discussion

This study selected some indicators according to WHO Core Drug Use Indicators for the rational use of drugs in developing country (15) and some researches in China to evaluate the effects of the NEMP on the rational use of drugs. Our results showed that the percentage of prescribed EML drugs rose significantly, the average numbers of drugs per prescription and average cost per prescription were declined significantly after the implementation of the NEMP. While the differences in the prescription proportion of antibiotics and injection were not statistically significant. The average number of drugs per prescription, the number of using antibiotics and hormone, regional differences (differences among economic developed city A, moderately developed city B, economic undeveloped city C), size of organization, sponsorship, business income, doctor degree, outpatient and emergency visits person times were important factors affecting the prescription costs.

### The role of the NEMP in promoting rational drug use

The NEMP plays a role in promoting rational drug use. The average numbers of drugs per prescription and average cost per prescription were declined significantly after the implementation of NEMP. The average numbers of drugs per prescription in our three pilot regions were 2.256, 2.890, and 3.234 compared with nearly 4 in Hubei Province (12) and 2.36 across 10 western China provinces (4). The reasons are as follow: on one hand, as the NEMP being implemented, primary care institutions only can provide suitable formulation, reasonable price essential drugs and implement “zero-profit drug policy”, the increased use of EML reduced the average cost per prescription; on the other hand, in order to implement the NEMP successfully, health administrative departments strengthen relevant training on the use of essential medicine. These lead to the decline of the mean of medicine varieties per prescription.

However, the role of NEMP in promoting rational drug use was limited. The average numbers of drugs per prescription, percentage of patients prescribed antibiotics and percentage of patients prescribed injections are important indicators for rational drug use assessed. The survey of WHO on community health agency in several countries in Asia and Africa showed that the mean of medicine varieties per prescription was 2.2 below (19), and recommended that other countries followed the index. Percentage of patients prescribed antibacterial drugs of WHO standard is 20.00%–26.80%, while in developing countries were about 27%∼63% and developed countries were about 10% (15, 20–22). WHO suggests the percentage of patients prescribed injections should be <10% (23). The average numbers of drugs per prescription in our study before and after the implementation of the NEMP were higher than the recommended value. In order to strengthen the rational antibiotics use, the countries around the world have developed different controlling policies. WHO advocated the principle for drug dosage forms was oral therapy instead of injection (24). Our study showed that percentage of patients prescribed antibiotics and injections were higher than the range recommended by WHO. It was closely related to the doctors’ prescribing habit and the aspirations of the patients affected the prescribing behavior through our interviewing with local doctors. Other studies in China also showed the same results (12, 25, 26).

### Factors affecting prescription costs

A few professions can be conducted in primary care institutions because of the technical level of physician, the medical equipment, and others were inferior to the large medical institutions, so income from prescribing, especially the drugs accounted the great proportion of the revenue structure of primary care institutions, and this leads to doctors’ prescribing behavior driven by economic interests. At the same time, the primary objective to implement the NEMP is to control the unreasonable rise in prescription costs, thus it is necessary to study factors affecting the cost of prescription (27, 28). We analyzed the influencing factors of the average cost per prescription by BP neural network. The average numbers of drugs per prescription, the use of hormones, antibiotics and two or more antibiotics reflect that the phenomenon of irrational drug use still existed in primary care institutions. However, this phenomenon has a certain influence on prescription costs, they were positively correlated with prescription charge, the prescription charges will be increased as the growth of the average numbers of drugs per prescription, hormones, antibiotics, and two or more antibiotics. Among these, an average number of drugs per prescription has the greatest impact on the prescription costs. These may be that the medical staff in primary care institutions had low medical technology and consciousness of rational drugs use, could not prescribe the drugs strictly according to the patients’ indication.

In addition, other factors of objective condition such as the doctor’s technology level, business income, geographical distribution, outpatient and emergency visit person times, the sponsorship, size of organization have a certain degree of impact on prescription cost. The doctor’s degree and the title level were positively related to prescription cost. In general, more patients undergoing difficult diseases would like to choose a hospital having advanced medical instrument equipment and a high level of technical personnel of medical institutions, this can explain why the doctor degree increase, the agency prescription costs will arise accordingly. The more of business income of primary care institutions was, the higher was the prescription costs, thus it also can illustrate that prescription costs were influenced by the behavior of the doctors pursuing high profit.

Prescription costs were affected by the geographical distribution in a certain extent, it may be that the primary care institutions of the three cities were different in economic development, resources, the living standards of residents and the doctor’s technical level. The outpatient and emergency visit person times and prescription costs were positively correlated, this accounted for the relatively better of the service scale and ability, so those medical agencies can attract the residents to see the doctor and buy medicines, especially the more of the complicated patients, prescription costs, therefore, will be higher. The different sponsorship, to some extent, affected the prescription costs in primary care institutions. This may be because primary care institutions held by the government and its subordinate medical institutions had the higher level of overall strength and services, primary care related policies were implemented more appropriate, and the service objects of those held by enterprises and institutions were more concentrated, service pertinence was stronger, so the doctor’s quality and consciousness of rational drug use were also relatively higher to the others. Size of organization on the effect of prescription cost was small, which on the one hand, showed that primary care institutions had begun to take shape, and do not need to increase the prescription cost and average number of drugs per prescription to maintain the body’s basic construction, on the other hand, infrastructure of primary care institutions has been basically completed, which embodies the fundamental role of the construction of the primary care system. In all, the average number of drugs per prescription has the greatest on the prescription cost and is important to control it.

Several limitations of the current study should be highlighted. Firstly, only the outpatient prescriptions were included without the inpatient prescriptions. Secondly, we did not study the other provinces. However, our study could provide some suggestions for the implementation of NEMP.

## Conclusion

The Chinese NEMP can promote the rational use of drugs in some degree, but its role is limited. We should not focus only on the EML and should make comprehensive NEMP, such as change the relation between the doctors and patients, enhance the medical personnel training about the rational use of drugs, implement the discerption of medical and drug for cutting off the benefit relation between medical institutions and drugs to improve the rational use of drugs.

## Ethical considerations

Ethical issues (Including plagiarism, informed consent, misconduct, data fabrication and/or falsification, double publication and/or submission, redundancy, etc.) have been completely observed by the authors.
